# A Highly Sensitive Flow Cytometric Approach to Detect Rare Antigen-Specific T Cells: Development and Comparison to Standard Monitoring Tools

**DOI:** 10.3390/cancers15030574

**Published:** 2023-01-17

**Authors:** Meytal Dror Levinsky, Baruch Brenner, Michal Yalon, Zohar Levi, Zvi Livneh, Zoya Cohen, Tamar Paz-Elizur, Rachel Grossman, Zvi Ram, Ilan Volovitz

**Affiliations:** 1 The Cancer Immunotherapy Laboratory, Tel Aviv Sourasky Medical Center, Tel Aviv 6423906, Israel; 2 The Neurosurgery Department, The Tel Aviv Sourasky Medical Center, Tel Aviv 6423906, Israel; 3 The Sackler Faculty of Medicine, The Tel Aviv University, Tel Aviv 6997801, Israel; 4 The Institute of Oncology, Davidoff Cancer Center, The Rabin Medical Center, Beilinson Hospital, Petach Tikva 4941492, Israel; 5 The Pediatric Hematology-Oncology Department, Safra Children’s Hospital, Sheba Medical Center, Ramat Gan 52621, Israel; 6 The Gastroenterology Department; The Rabin Medical Center, Petach Tikva 4941492, Israel; 7 The Biomolecular Sciences Department, The Weizmann Institute of Science, Rehovot 7610001, Israel; 8 The Felsenstein Medical Research Center, The Rabin Medical Center, Petach Tikva 4941492, Israel

**Keywords:** cancer, peptide vaccine, RNA vaccine, personalized cancer vaccine, cancer testis antigens, neoantigens, self-antigens, flow cytometry, ELISpot, T cells

## Abstract

**Simple Summary:**

Personalized anti-cancer vaccines utilize peptides derived from mutated proteins (neoantigens) or from proteins aberrantly expressed by the cancer cells (e.g., cancer testis antigens). Vaccination increases the frequency of anti-tumoral T cells and promotes their antigen-specific activation. Current methods used for identifying peptides which have elicited T-cell responses, do not measure these responses with sufficient sensitivity. Non-antigen-specific background activation frequency of currently applied methodologies ranges from 0.1–0.7%, while the frequencies of antigen-specific responses to vaccinated peptides are often at the lower end of this range (0.05–0.2%). As a result, many peptides which have succeeded in eliciting a response are not detected. We developed a methodology that can identify response frequencies as low as 0.01–0.02%, thereby facilitating the detection of T-cell responses that would otherwise be missed. Our method enables more accurate monitoring of the dynamic changes in vaccination-induced T-cell responses and thereby more efficacious design of personalized vaccines.

**Abstract:**

Personalized vaccines against patient-unique tumor-associated antigens represent a promising new approach for cancer immunotherapy. Vaccine efficacy is assessed by quantification of changes in the frequency and/or the activity of antigen-specific T cells. Enzyme-linked immunosorbent spot (ELISpot) and flow cytometry (FCM) are methodologies frequently used for assessing vaccine efficacy. We tested these methodologies and found that both ELISpot and standard FCM [monitoring CD3/CD4/CD8/IFNγ/Viability+CD14+CD19 (dump)] demonstrate background IFNγ secretion, which, in many cases, was higher than the antigen-specific signal measured by the respective methodology (frequently ranging around 0.05–0.2%). To detect such weak T-cell responses, we developed an FCM panel that included two early activation markers, 4-1BB (CD137) and CD40L (CD154), in addition to the above-cited markers. These two activation markers have a close to zero background expression and are rapidly upregulated following antigen-specific activation. They enabled the quantification of rare T cells responding to antigens within the assay well. Background IFNγ-positive CD4 T cell frequencies decreased to 0.019% ± 0.028% and CD8 T cells to 0.009% ± 0.013%, which are 19 and 13 times lower, respectively, than without the use of these markers. The presented methodology enables highly sensitive monitoring of T-cell responses to tumor-associated antigens in the very low, but clinically relevant, frequencies.

## 1. Introduction

Personalized vaccines designed to target the patient’s tumor-associated antigens (TAA) are one of the most promising avenues of current cancer immunotherapy [1,2]. Such vaccines are comprised of shorter (8–10 AA) or longer peptides (>12 AA) predicted to bind the patient’s unique set of HLA class I or HLA class II molecules, respectively [1,3]. Alternatively, the antigens can be encoded as RNA vaccines [2]. T cells activated by these antigens proliferate, increase their clonal relative frequency and mature in terms of their functional capabilities (cytotoxicity and cytokine secretion, etc.) [4].

Most current personalized peptide/RNA vaccines target neoantigens [1,2]. Neoantigens are potent tumor rejection antigens, with no on-target off-tumor effects. While this represents a suitable therapeutic approach for tumors of high tumor mutational burden (TMB), many cancers are low in TMB [3] and have only a handful of actionable neoantigens [3,5]. For instance, HLA peptidomics performed on plasma and tumor samples from human TMB-low glioblastoma (GBM) samples have not identified any neoantigens but identified numerous cancer testis antigens (CTAs) [3,6]. CTAs are antigens expressed by tumor cells but not by adult somatic tissues. They have low or no on-target off-tumor toxicities [1,7]. Attempts to target gliomas with neoantigen-based vaccines have thus far not yielded therapeutically significant results [6]. Thus, in TMB-low, non-virally-induced tumors, CTAs may represent promising tumor-associated antigens [1,8].

Irrespective of the targeted antigens, assessment of vaccine immunogenicity is usually based on quantification of the temporal changes in the frequency and/or the activity of antigen-specific T cells throughout treatment. These changes were shown to correlate with treatment efficacy [1,2,9,10]. Antigen-specific responses are defined as those where peptide-specific responses to the vaccinated peptide are higher than responses to a negative control peptide (or no peptide). Preferably, the response to the vaccinated peptide, following vaccination, is also higher than the response to that peptide before vaccination (assuming no spontaneous anti-peptide response had occurred).

Frequencies of pre-vaccine T cells responsive to tumor antigens in melanoma patients (a highly immunogenic tumor) range from 10^−5^ to 10^−3^ [2,6,11]. Following vaccination, these frequencies could rise to 1–5 × 10^−4^ when a peptide vaccine was used [1] or to 2 × 10^−4^ – 5 × 10^−2^ when an RNA vaccine was used [2]. Given such low and variable pre- and post-vaccine T cell frequencies, clinically relevant assays must display a very low pre-activation background, optimally less than the pre-vaccine antigen-specific responses.

Several approaches have been developed to assess vaccine-induced functional changes in the frequency of responsive T cells. These assays usually measure IFNγ secretion on the single-cell level. IFNγ, a hallmark Th1 cytokine, and Th1 polarization, were repeatedly shown to correlate with effective anti-cancer immune responses and with better prognosis [12]. The most widely used methods to detect anti-tumor responses are enzyme-linked immune absorbent spot (ELISpot) and flow cytometry (FCM). ELISpot detects individual cytokine-secreting cells following stimulation with a specific antigen [13,14,15,16]. It is widely used by academic and industrial laboratories due to its simplicity of use, and relatively simple validation, harmonization and quality assurance [17]. Critically, the IFNγ, monitored by ELISpot, can be secreted by CD4 and CD8 αβ-T cells, but also secreted by γδ-T cells, natural killer (NK) cells, natural killer T cells (NKT), B cells and various antigen-presenting cells (APC) [18], thereby limiting the assay’s specificity.

Another commonly used monitoring methodology is FCM, which can rapidly query large numbers of parameters per single cell in a large number of queried cells. These parameters may include cell identity (e.g., CD3/4/8), cell viability, the expression of extra- and intracellular activation or function-related markers (e.g., IFNγ and PD1), TcR specificity (using tetramers) and T-cell maturation markers (e.g., TCF1 and CCR7) [14,19,20,21]. The ability to simultaneously measure several parameters is a significant advantage of FCM over ELISpot which usually analyzes a single parameter [22]. The ability to concurrently quantify several parameters on the single cell level also enables monitoring of complex parameters, such as T cell polyfunctionality—the ability of a single cell to concurrently respond to an antigen by several functions. Polyfunctionality was shown to correlate with more effective clinical responses to viruses or cancer [23].

While having many merits, FCM requires considerably higher technical skills and more expensive instrumentation. Due to its technical complexity, it is more limited in its capacity for harmonization and technical validation. The positioning of gates in FCM is subjective to some extent, especially with continuous (non-dichotomic) parameters, such as IFNγ, making quality assurance steps for FCM more complicated compared to ELISpot [22,23].

Though a handful of clinical trials that monitored vaccine efficacy used both ELISpot and FCM [1,2], there has not yet been any systematic investigation of the relative strengths and weaknesses of these two methodologies or evaluation which one, if either, should be chosen to monitor cancer vaccination trials.

Here, we used three biological sample sets to compare FCM with ELISpot. We found that both methodologies, used standardly, may miss the identification of many, and in some cases most, of the responding peptides. We then went on to develop a methodology that could more accurately detect rare cells responding to cancer vaccines or self-antigens. Our eight-color FCM panel combines the identification of T cells by CD3/CD4/CD8, the removal of unwarranted B cells, monocytes and dead cells, the detection of two pivotal antitumoral functionality markers (IFNγ and CD107a) [24,25] and the addition of two early activation markers (4-1BB and CD40L) [26] used together to detect responding CD4 or CD8 T cells. These features, in combination, enable the identification of recently activated T cells that have responded to peptide activation during the overnight in-vitro assay. The addition of the selected early activation markers reduced background activation noise by more than 10-fold to approximately 1–2 × 10^−5^, enabling the detection of T-cell responses that were otherwise undetectable by either ELISpot or by standard FCM.

## 2. Materials and Methods

### 2.1. Human Samples

A pediatric patient (male, 8 years old) diagnosed as having diffuse hemispheric glioma (H3 G34-mutant) was administered compassionate use treatment with vaccine peptides designed to target the antigens expressed by his tumor, at the Tel-Hashomer Sheba Medical Center. The vaccination protocol included three sets of vaccines (S1–3), each comprised of ten different peptides (P1–P30). Approximately 20 mL of blood was collected from the patient in each sampling. Blood sampling was carried out before each peptide vaccination (day 0), on days 9–14 post-vaccine (early response), and on day 21 (late response). It was also performed on days 6–11 following the first booster vaccine per each vaccination set (see blood sampling protocol in Figure S1).

Blood samples were also collected from four patients with colorectal cancer (CRC) and four healthy donors (HD) following colonoscopy with no significant findings. The CRC and HD samples were obtained at the Rabin-Beilinson Medical Center (IRB approval number 0773-20-RMC) (see blood sampling protocol in Figure S2).

Peripheral blood mononuclear cells (PBMC) were purified from blood samples using the buffy layer method using Lymphoprep (Serumwerk Bernburg AG, Germany) and frozen before use. The glioma samples were kept in a −80 °C freezer and used within 1–2 months of acquisition. HD and CRC samples were kept in liquid nitrogen.

All experimental procedures were performed at the Tel-Aviv Sourasky Medical Center. Informed consent was obtained from all subjects involved in the study or from their legal guardians.

### 2.2. Glioma Vaccine Peptides and CRC-Related Peptides Used for Immune Monitoring

The glioma patient was vaccinated with three sets of 10 predicted tumor-specific peptides [e.g., peptide 13 from set 2 is denoted as S2P13 (Table S1)], many of the antigens were CTA (e.g., KDM5B, TSSK2), some were neoantigens (e.g., H3-3A G35R, TP53 R248Q) or other TAAs. The 9mer peptides were predicted to be restricted by the patient’s HLA- class I alleles: A3001/A6601, B0705/B3801, C1203/C1505. The 12-27mer peptides were predicted to be restricted by the patient’s HLA class-II alleles: DRB11101, DQA10104/ DQA10505, DQB10301/ DQB10503, DPA10103/DPB10401. The peptides were dissolved in 1% DMSO at 20 mg/mL, aliquoted and stored at −20 °C. Before use, the peptides were diluted in assay medium, X-VIVO15 serum-free-defined medium (Lonza Basel, Switzerland) and filtered with a Puradisc-13 sterile 0.2 μm PVDF syringe filter (Whatman, Cytiva, Maidstone, UK).

### 2.3. CRC Peptide Pools and Assay Controls

Peptide pools (PepMixTM (PM)—JPT Peptide Technology, Berlin, Germany) were used to evaluate T-cell responses to the CRC and the control antigens. The peptide pools consisted of Mucin -1 (MUC1) or carcinoembryonic antigen (CEA)—both of which are CRC-related non-mutated antigens [27]. Actin peptide pool was used as a negative-control self-antigen. The pools consisted of multiple 15mer peptides spanning the entire protein with 11 aa overlaps. The CEFT positive-control peptide pool is composed of 27 peptides specific to several widespread pathogens (CMV, EBV, influenza and *Clostridium tetani* toxin) restricted by the most abundant human HLA alleles. The peptide pools were dissolved in 1% DMSO, aliquoted, stored at −80 °C (for long-term storage) and used at 1 μg/mL per individual peptide.

### 2.4. T-Cell Polyfunctional Assay

T-cell activation and polyfunctionality assays were performed by culturing PBMC (in average 4.6 × 10^5^ cells, depending on sample availability) with single peptides (the glioma vaccine) or with various peptide pools (CRC patients or HDs). Assays were performed in V-shaped 96-well plates (Corning Incorporated, Kennebunk, ME, USA) incubated overnight at 37 °C in X-VIVO15 medium. PBMC were stimulated with 1ug/mL staphylococcal enterotoxin B (SEB) (Sigma-Aldrich, Saint Louis, MO, USA) as a positive control. Negative controls in all assays were PBMC incubated with assay medium only (no peptides). Five hours before harvesting, microcultures were supplemented with fluorochrome-conjugated CD107a Ab (Biolegend, San Diego, CA, USA), 0.07% Golgistop (BD Biosciences, Franklin Lakes, NJ, USA), 0.1% brefeldin A (Sigma-Aldrich) and 1 μg/mL CD28/CD49d (Fastimmune, BD Biosciences). Fastimmune was used to provide optimal co-stimulation, facilitating the detection of rare antigen-specific cytokine-producing cells. All the cells were then harvested and collected for FACS staining and analysis. The cells were stained for viability by using violet fixable LIVE/DEAD amine viability dye (ViViD-L34955; Thermo Fisher Scientific, Waltham, MA, USA), and then extracellularly stained for CD8, CD14 and CD19. The CD3, CD4, IFNγ, 4-1BB and CD40L [26] markers were stained intracellularly following fixation/permeabilization with the Fix/Perm Kit (BD Biosciences) according to the manufacturer’s protocol [21]. CD3 and CD4 were stained intracellularly since T cell activation drives their internalization. The cells were analyzed by FCM on a 3-laser Canto-II (BD Biosciences) analyzer. A median of 4.34 × 10^4^ cells were acquired per 196 samples; in 96.5% of these samples, more than 1.1 × 10^4^ cells were acquired. Cell losses occur due to cell death in overnight incubation, the serial staining procedure and incomplete sample acquisition (to avoid air clogging). Analysis was performed with the FlowJo analytical package (BD Biosciences).

### 2.5. ELISpot-Like Flow Cytometry (ELF) Analysis

To simulate the ELISpot approach by means of FCM data, all cell types identified by FCM were gated for IFNγ secretion. The FCM-identified cell groups were: live CD4 T cells, live CD8 T cells, live non-CD3 cells (e.g., NK or NKT cells) and cells in the dump channel, which included live and dead B cells, live and dead monocytes and other dead cells.

### 2.6. ELISpot Assay

Four samples, including two PBMC samples from the pediatric glioma patient, one HD PBMC sample and one CRC PBMCs sample, were analyzed by FCM and in parallel, by ELISpot (DIACLONE SAS, Besançon Cedex, France) according to the manufacturers’ protocols. The ELISpot plates were treated with 35% ethanol, washed and coated by an IFNγ capture antibody overnight at 4 °C. The plates were then blocked with assay medium, after which 1.1 × 10^5^ cells were cultured with relevant peptides or peptide pools overnight at 37 °C in X-VIVO15 medium. Following the incubation period, the cells were washed, and the plates were treated with FITC-conjugated anti-IFNγ detection antibodies and then with anti-FITC horseradish peroxidase-conjugated antibodies. The AEC (3-amino-9-ethylcarbazole) substrate-containing-buffer generated the IFNγ spots. A microscopic camera (Discovery-VMS-001) with the MicroCapture Veho-VMS-004 software (Veho, Hampshire, UK) was used to micrograph the assayed wells. The FIJI ImageJ2 processing package was used to analyze the micrographed spot-forming units (SFU).

### 2.7. Defining Assay Sensitivity Using Background Noise—Theoretical Considerations

Random measurement noise derives from either natural variation in the assayed cells (i.e., background activation) or from noise in the detection methodology (i.e., assay precision). Defining what constitutes a “response” is a matter of controversy. Horton et al. defined positive (“responding”) wells in intracellular cytokine secretion (ICS) assays as wells in which the cytokine-secreting cell frequency was at least three-fold higher than the negative control and at least 0.05% above the background. Roederer [28] argued against an artificial positivity threshold and suggested that negative and positive events/wells can only be defined by comparison of the measurement against a set of negative and positive control samples. Furthermore, in specific contexts, even a single event can define a “positive” well. Roederer’s approach is both theoretically and practically more applicable to rare cell detection than Horton’s. Nonetheless, both approaches define an assay’s lower limit of detection, or sensitivity, by the mean and by the distribution of the background noise (e.g., response to no-peptide or to negative control peptides). We found it is advisable to run at least a duplicate of the no-peptide group in order to obtain time-point-specific background + standard deviation (STD), although this was not performed in all our assays. See Section 3.7, the section discussing the definition of a responding peptide, for further details.

## 3. Results

### 3.1. A Variety of Cells, including T Cells, Secrete IFNγ

Peptide vaccines aim to activate both CD4+ and CD8+ T cells. While FCM can specifically identify live CD4+ and CD8+ T cells, ELISpot detects IFNγ secretion by any cell secreting IFNγ– these include those targeted by vaccination (i.e., T cells) and other cells, not targeted by vaccination as B cells, monocytes, various CD3- cells, CD3+ non-αβ-T cells [18,29] and dead cells, non-specifically binding the IFNγ Ab.

To investigate the extent to which ELISpot detects IFNγ secretion by non-αβ-T cells, we compared IFNγ secretion by FCM either by fully-gated live (ViViD^dim^) CD4+ or CD8+ T cells (CD3^+^CD14-CD19-) or secretion by vaccination-untargeted cells: live CD4^−^CD8^−^ T cells (mostly γδ), live CD3-CD14-CD19- cells (such as NK, NK-T cells, etc.) and monocytes, B cells, and/or dead cells (all on a single dump channel).

**Figure 1:**
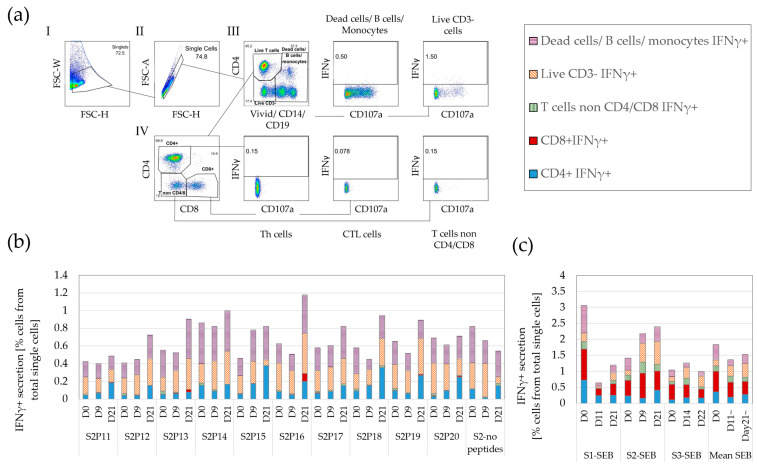
Vaccination-targeted and -untargeted secretion of IFNγ in ELISpot-like flow cytometry (ELF): PBMC from a glioma patient were cultured with or without tumor-specific peptides overnight and stained with a full FCM panel (see text). (**a**) Gating strategy of IFNγ secretion to tumor-specific peptides. The cells were analyzed by FCM and serially gated for (I+II) non-doublet/clumped single-cell gates; (III) live T cells (using a wide gate to include all activated T cells), live CD3- cells, and B cells / monocytes /dead cells (on a single dump channel); (IV) grouping into CD4+/ CD8+/ CD4-CD8- T cells. All cell populations were then gated for IFNγ secretion. Repeated 6 times using different sample sets. (**b**) Total IFNγ secretion to peptides (P11–20) in vaccine Set2 (S2) displayed by the cell subsets secreting IFNγ. (**c**) IFNγ secretion in response to SEB displayed by the cell subsets secreting IFNγ.

Figure 1a depicts the gating strategy used to identify IFNγ secretion from both vaccination-targeted and untargeted cell populations. This emulates the signal that would be detected by ELISpot, using FCM data [heretofore termed ELISpot-like-flow (ELF)]. Within the summed IFNγ secretion by all cell types secreting IFNγ, the contribution of non-CD4 or non-CD8 T cells ranged from 15–84% of the total signal (Figure 1b). Background IFNγ secretion (no-peptide group) measured in ELF 0.8% ± 0.62%, from which 0.4 ± 0.22% (~50%) stemmed from vaccination-untargeted cells. These background IFNγ secretion levels are considerably higher than most post-vaccine responses [1,2]. ELF for IFNγ secretion displayed a similar high and variable background in response to no peptides in PBMC obtained from a CRC patient (0.72% ± 0.18%) and a HD (0.07% ± 0.01%) (Figure S3).

To assess whether non-CD4+/CD8+ T cell-derived IFNγ secretion is found only in response to weak antigens or no antigens, we assessed IFNγ secretion to SEB, a strong polyclonal T-cell activator. Figure 1c demonstrates that non-CD4+/CD8+ T-cell responses represented approximately one half of the FCM-detected IFNγ response to strong polyclonal antigens. In summary, a significant portion of the IFNγ signal measured by ELISpot both in the measurements of signal and of noise may be derived from secretion by vaccination-untargeted cells which are neither CD4 nor CD8 T cells.

### 3.2. IFNγ ELISpot Demonstrates a Low but Highly Variable Background

**Figure 2:**
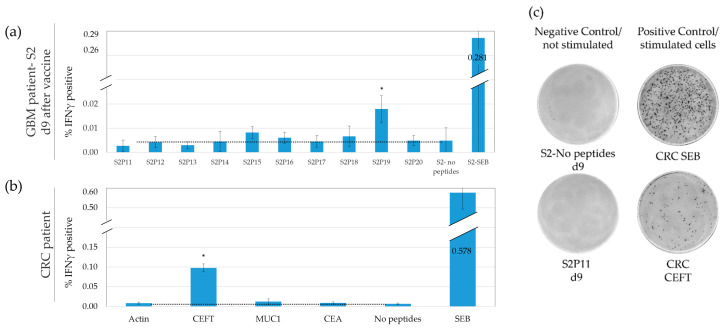
Monitoring responses to vaccinated peptides and to peptide pools using ELISpot: PBMC samples obtained from a glioma patient, 9 days after vaccination with set 2 vaccine (**a**) and PBMC from a CRC patient (**b**) were cultured with or without peptides/peptide pools, with the positive control CEFT peptide pool or with the SEB superantigen. Spot-forming units (SFU) were micrographed and quantified. (**a**) Mean and STD of triplicates from glioma vaccine set 2, (**b**) mean and STD of triplicates from a CRC patient. A dotted reference line was drawn from the no-peptides groups. An asterisk represents a response that is significantly different from the no peptides group (t-test, P < 0.05). (**c**) Representative micrographs of IFNγ ELISpot: positive control SEB and CEFT; response to specific peptides and to negative control-no peptides.

ELISpot is widely used to monitor responses to tumor antigens [1,6]. To evaluate ELISpot’s background and precision, we assayed two PBMC samples from the glioma patient (samples from which we had excess material on day nine, (Figure 2a) and day 21 following vaccination with the second set of peptides). We additionally monitored a CRC patient’s PBMC (Figure 2b) and a HD’s PBMC (not shown). These four samples were monitored in parallel by FCM and by ELISpot (triplicates per each antigen, 1.1 × 10^5^ cells per ELISpot-well)—see representative micrographs in Figure 2c.

The ELISpot triplicates demonstrated low numbers of positive cells. The mean SFU per responding well was 8.5 SFU. With such low numbers, the assay precision should be strongly affected by the statistical variation of the observed numbers of responding cells, around the expected cell numbers (i.e., “true” response frequency) [28]. The numbers of observed positive events (i.e., SFUs) comprise discrete and independent events that are Poisson distributed with Mean = Variance = λ [30]. A high variability was observed within triplicates with a mean coefficient of variance (CV = mean/STD × 100) of 45% for all no peptide-stimulated and peptide-stimulated wells. The larger part of the CV (78% of the 45% observed CV) can be attributed to the Poisson distribution of rare events (expected CV = λ/√λ × 100 = 8.5/√8.5 × 100 = 35%).

This large inbuilt statistical variance of rare-cell detection strongly impacts the ability of any assay to detect low-frequency responses. Indeed, only one peptide from set two (S2P19) analyzed on day nine post-vaccine was significantly different from the no peptide (negative) control (*P* < 0.05 *t*-test). Note that most CTAs and neoantigens produce subtle responses [22,30], thus many responses are expected to be missed by ELISpot due to the high variability, albeit low background.

In comparison to the response to weak peptide antigens, the common viral antigens found in the CEFT peptide pool (influenza, EBV, and CMV [25]) or the SEB superantigen stimulated stronger IFNγ secretion. Responses to these strong antigens demonstrated a mean of 442 SFUs per well. The observed mean CV for the stronger antigens was only 10.7%. As expected, assay precision, in this case, was dominated by technical inconsistencies (56% of the observed CV), more than by the Poisson distribution of positive events (44% of the observed CV). In summary, when measuring weaker responses, a large portion of assay noise arises from a non-biological statistical noise [30].

### 3.3. Low Correlations between ELISpot and FCM in the Weaker T-Cell Response Range

**Figure 3:**
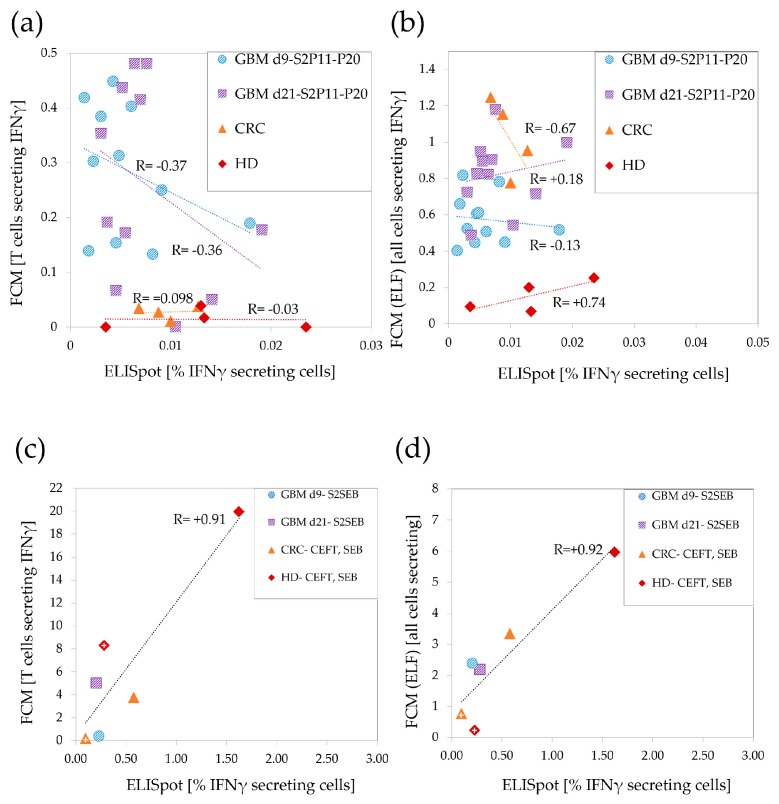
Correlation between ELISpot-measured responses to parallel FCM-measured ones: Responses to peptides in set 2 glioma vaccine (set2 peptides 11–20) in two time points (d9, d21), and responses to peptide pools in CRC and HD samples analyzed by FCM and ELISpot. (**a**) Summed CD4+ and CD8+ T-cell responses (FCM) correlated to ELISpot SFU frequencies. (**b**) All IFNγ+ responsive cells (FCM-ELF) correlated to ELISpot SFU frequencies. FCM (**c**) and FCM-ELF (**d**) correlations to ELISpot used to monitor responses to stronger antigens: CEFT and SEB. For each set of samples, Pearson’s correlation coefficient (R) was calculated, and a trend line was added.

To evaluate the correlation between IFNγ secretion as measured by either ELISpot, ELF or by using FCM, we used the data from the four sets of samples evaluated in parallel by FCM and ELISpot (CRC, HD, and two glioma samples from different time points). The samples were stimulated identically for each assay. Figure 3 shows that neither ELF (i.e., all IFNγ+ cells measured by FCM) nor the summation of IFNγ secretion by CD4+ and CD8+ T cells (denoted FCM) correlated with the ELISpot SFU frequencies in the weaker T-cell response range (Figure 3a,b). Importantly, while the dynamic range for IFNγ-secreting T cells in FCM was 0.01–0.48%, and in ELF at a slightly higher range (0.05–1.24%), all of the ELISpot cultures responded below 0.03%. This suggests that ELISpot detects only the cells most strongly secreting IFNγ.

In contrast to the lack of correlation found for the weak T cell responses, very strong correlations were found between ELISpot and ELF (R = +0.92) or between ELISpot and the summed CD4- and CD8- T-cell responses in FCM (R = +0.91) for the stronger antigens (CEFT and SEB) (Figure 3c,d), supporting the fact that these methodologies indeed measure the same signal. Again, here, the dynamic range of the responses monitored by ELISpot was 4–10 times lower than that detected by FCM.

Our data indicate that ELISpot is in correlation with FCM only in frequency ranges stronger than commonly expected for most cancer antigens (>0.2%). Moreover, the reduced sensitivity of ELISpot (4 to 10 times lower in our sample sets) further exacerbates the negative effects of non-biological statistical noise that considerably limits assay sensitivity. 

### 3.4. IFNγ Background in Standard FCM Is High and Variable, Impeding Accurate Detection of Rare Responding T Cells

**Figure 4:**
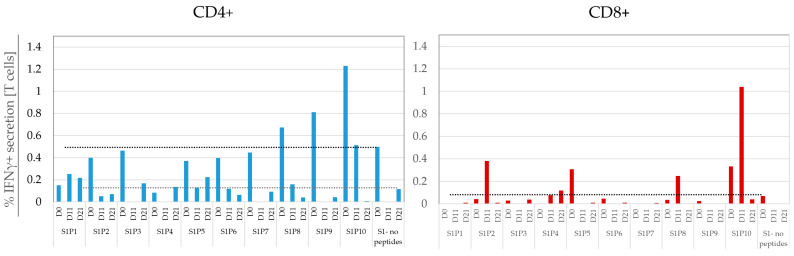
FCM detection of IFNγ secretion to vaccine-peptides: A glioma patient’s PBMC were cultured with or without vaccine peptides overnight and stained by means of a T-cell polyfunctional panel. Bar graphs show IFNγ secretion of CD4+ and CD8+ T cells to Set 1 (S1) peptides. Repeated with 3 different sets of samples with similar results. A dotted reference line is drawn from the non-zero no-peptides groups.

Accurate monitoring of CD4+ and CD8+ T-cell responses to tumor antigens is critical for the development of effective vaccines [31]. We used a high-end “standard” FCM panel, comparable to Lamoreaux et al. [21], to monitor responses to peptides or peptide pools. The panel identified live CD4+ or CD8+ T cells (CD3+CD14−CD19−ViViD^dim^) and evaluated IFNγ secretion to no peptides and to the vaccine peptides (Figure 4). The background (no peptides) IFNγ responses, summed from all three sets of the glioma patient’s PBMC, measured 0.78% ± 1.05% for CD4+ T cells and 0.016% ± 0.02% for CD8+ T cells. A similar high or variable background IFNγ secretion was found in the response of PBMC to no peptides from the four CRC patients (0.14% ± 0.21% for CD4 and 0.19% ± 0.35% for CD8 T cells) and in the four HD blood samples (0.05% ± 0.05% for CD4 and 0.09% ± 0.16% for CD8 T cells) (not shown).

The utilized FCM assay protocol enables the measurement of T cell background IFNγ noise, which is not attributable to secretion by non-T cells or related to artifacts as non-specific Ab binding by dead cells. Notwithstanding, high and variable background IFNγ secretion by live T-cells was observed, more strongly in CD4 T cells but also in CD8 T cells. This background secretion is likely attributable to activation of T cells that had occurred prior to the in-vitro culture with antigen/s. This high background noise impedes the ability to sensitively detect reported post-vaccine responses to tumor antigens that start as low as 0.01–0.02% [2,6].

### 3.5. Inclusion of Early Activation Markers Enable the Detection of Weak T-Cell Responses by Significantly Reducing Assay Background

To reduce antigen non-specific activation background, we incorporated into our panel two early activation markers, 4-1BB (CD137) and CD40L (CD154), gated together using a logical ‘OR’ gate. The gating on either of these activation markers preceded any functional analysis in the serial gating strategy, to enable the detection of T cells which were activated within the assay well. This discriminates the identified cells from T cells expressing a functional marker (e.g., secreting IFNγ) due to pre-assay activation.

While 4-1BB is mainly used as a marker for activated CD8+ T cells [32], activated CD8+ T cells may also express CD40L enabling them to execute immunologic helper functions [19,33]. Similarly, while CD40L is mainly used to identify antigen-activated CD4+ T cells [34], activated CD4 T cells may also upregulate 4-1BB upon activation [19,35]. CD40L and 4-1BB have negligible expression in cells that have not been recently activated [36,37] and they are both upregulated during the ~20-h duration of the assay [19].

Our panel included several other components aimed at increasing sensitivity by reducing the non-specific background. We used a fixable amine viability marker, for removal of dead cells which non-specifically bind antibodies in functional T-cell assays [38]. We also removed vaccine-untargeted cells, such as B cells and monocytes, that may also secrete IFNγ [18,21] and, as shown above (Figure 1b,c), likely contribute to the IFNγ signal. Additionally, B cells and monocytes may nonspecifically bind antibodies directed to other cells or markers [21].

In addition to IFNγ, we also included the CD107a functional marker [25]. CD107a is a glycoprotein found in the luminal surface of cytotoxic granules that is exposed on the cell membrane upon cytotoxic granule degranulation [25,39]. While CD107a is a widely used functional marker for CD8+ cytotoxic T lymphocytes (CTL) [25], some CD4+ T cells were shown to upregulate CD107a and secrete granzyme B [40,41]. Notably, the designed panel follows only Th1- and cytotoxicity-oriented responses (usually correlated with better responses in cancer). Other cytokines associated with additional Th polarizations (e.g., IL17A, IL4 and TGFβ) were not monitored.

**Figure 5:**
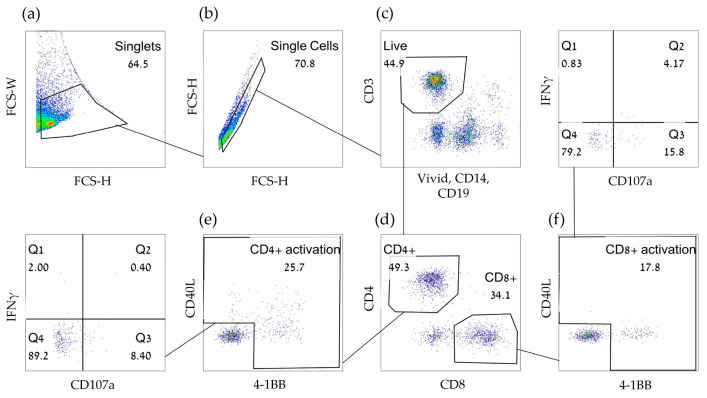
Full gating strategy for recently activated T cells using the Activation marker-inclusive FCM (A-FCM)**.** PBMC were cultured with or without peptides overnight and stained using a panel containing, among others, antibodies to 4-1BB, CD40L, IFNγ and CD107a. The cells were analyzed by FCM and serially gated for (**a**,**b**) Non-doublet/clumped single-cell gates. (**c**) Live CD3+ T cells which do not express CD14 or CD19. (**d**) T-helpers (CD4+) and cytotoxic T cells (CD8+). (**e**,**f**) T cells expressing one or more activation markers. Each population was then gated for degranulation (CD107a) and for IFNγ secretion.

**Figure 6:**
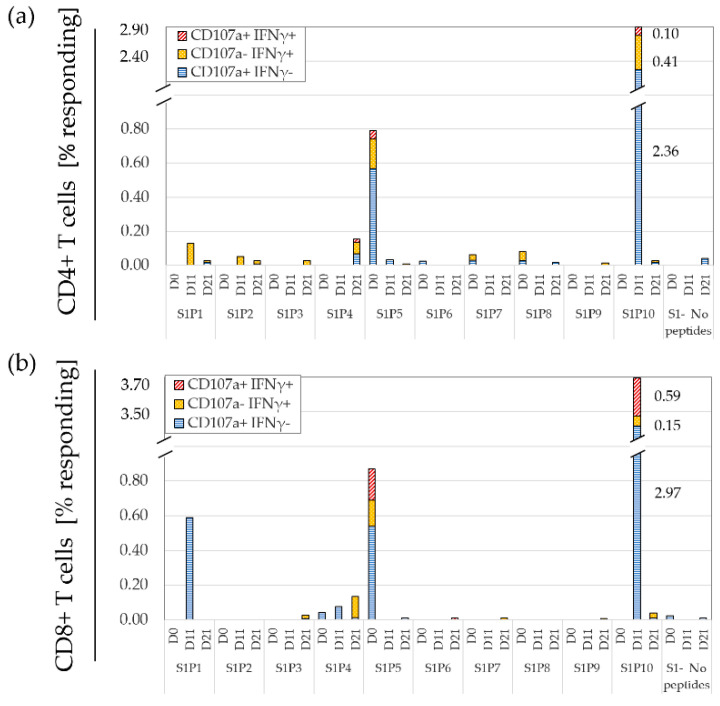
The use of early activation markers enables detection of weak responses to peptides. A glioma patient’s PBMC were cultured with or without peptides overnight then stained and analyzed using the Activation marker-inclusive FCM approach (A-FCM). Shown are mono- and polyfunctional responses (IFNγ/CD107a) of CD4 (**a**) and CD8 (**b**) T cells responding to set 1 peptides. These T cells have also expressed one or more early activation markers - 4-1BB or CD40L (representative results).

Figure 5 depicts a gating strategy utilizing all panel components (henceforth referred to as activation marker-inclusive FCM: A-FCM). Figure 6 shows the polyfunctional responses (IFNγ and CD107a) to set-1 vaccine peptides. Importantly, background IFNγ responses with A-FCM to no peptides were 0.019% ± 0.028% for CD4+ cells and 0.009% ± 0.013% for CD8 T cells in the glioma vaccine sets (*n* = 4). These background levels for IFNγ were 19 and 13 times lower than the FCM IFNγ background levels without the utilization of early activation markers for CD4 and CD8 T cells, respectively. With such low background responses, A-FCM could easily detect many antigen-specific CD8 T-cell responses that measured in average of 0.09% (the mean of all non-zero responses in the set of responding peptides post-vaccination—see next section) or 0.16% for CD4+ T cells (measured similarly). Comparably low background IFNγ background responses were detected to no peptides in the CRC patient cohort (CD4+ 0.01% ± 0.01% and CD8+ 0.02% ± 0.05%, *n* = 4) and the HD cohort (CD4+ 0.004% ± 0.01% and CD8+ 0.004% ± 0.01%, *n* = 4).

An example of the ability of the assay to reduce non-specific background is the Actin, peptide pool, used as a negative control with the CRC patients and the HDs. The A-FCM approach, removed the high pre-assay activation background, demonstrating average net responses to Actin ranging from 0.01–0.03% (close to the assay’s noise level - see Figure S4). Without the use of early activation markers, standard FCM demonstrated Actin responses that were 12–27 times higher for CD4+ and 5–30 times higher for CD8+ T cells compared to those measured using A-FCM. Similar range of background IFNγ responses to Actin were also recorded by others [42]. Such high background responses to self-antigens using standard FCM likely represent pre-assay antigen-unrelated responses, (as seen in Figure 4).

The addition of the CD107a to the panel enabled the identification of more responding peptides (two additional CD8 and two additional CD4 peptides per 30 peptides—see next section). CD107a demonstrated, using standard FCM, a considerably higher pre-activation background compared to IFNγ, especially in CD8+ T cells (CD8+ 0.085% ± 0.132%, CD4+ 0.065% ± 0.055%, *n* = 4). Note that the background responses for A-FCM presented above were calculated for cells that were positive for either IFNγ or CD107a or both.

To evaluate the methodology’s inter- and intra-assay reproducibility, we ran A-FCM on HD PBMC sample at two different time points in triplicates. In this preliminary validation/reproducibility assay we found that the triplicates showed very small variation (mean STD = ±0.015%). The differences between the means of each two repeats per the same antigen ran at different time-points were also small (mean of the absolute difference = 0.027% (Table S2).

Taken together, our data demonstrate that the A-FCM approach may enable the detection of responses to very weak tumor antigens and possibly self-antigens that are otherwise below noise level. The low and reproducible “distribution of negatives” [28] strongly powers the assay’s ability to detect weak responses as real responses.

### 3.6. Monitoring the Temporal Dynamics of Peptide Responses

To evaluate extended temporal changes (more than 21 days) in the glioma patient’s T-cell responses, we also included responses to the booster vaccines. PBMC were collected 6 to 11 days after each booster vaccination (expecting an earlier recall response) and analyzed using the A-FCM protocol. Three possible antigen-responsive cell groups were expected as each sample set underwent two types of analyses evaluating T cell activation (4-1BB and CD40L) and functional responses (IFNγ and CD107a): (1) Cells expressing early activation markers only but not IFNγ/CD107a. These may represent activated cells responding to antigens in the well by a non-Th1/cellular response. (2) Cells which are functional (IFNγ/CD107a positive) but not positive for any activation markers. These likely represent cells functionally activated prior to the assay. (3) Recently activated cells which are also functional. These represent the population of cells responding to a specific antigen within the well by a Th1/cytotoxicity-related pattern.

**Figure 7:**
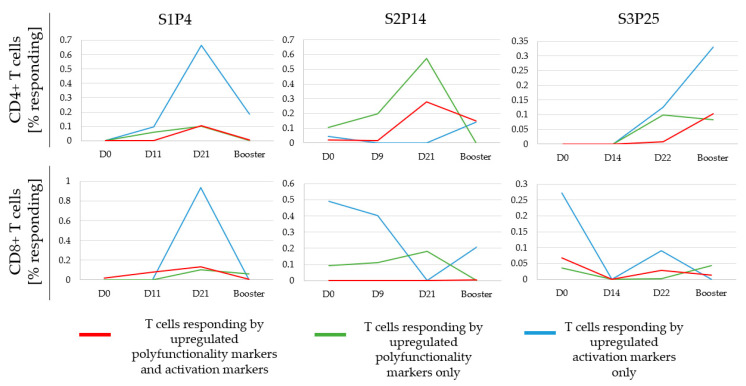
Effect of peptide vaccines and boosters on T-cell activation and functional responses. A glioma patient’s PBMC were cultured with or without peptides overnight, then stained and analyzed using the activation marker-inclusive FCM (A-FCM) protocol. T cells were monitored for early activation markers (4-1BB and CD40L) and for polyfunctionality (IFNγ and/or degranulation-CD107a). Shown are CD4+ helper T-cell (Th) and CD8+ cytotoxic T-cell (CTL) response dynamics for three representative peptides, one from each set.

Figure 7 depicts CD4 and CD8 responses showing several different response dynamics. Depicted are the “net” functional responses, i.e., peptide response minus the no peptide background. It reveals that the booster vaccine had increased the extent of T-cell responses in some cases (S3P25), but not in others (S1P4).

Differential responses to boosters were also observed following a second dose of COVID vaccine. T cells from infection-naïve patients usually secreted more IFNγ to vaccine-related peptides following the second vaccine (booster). Differently, T cell responses in convalescents either increased, decreased or did not change after receiving the booster. Vaccination of a cancer patient with different tumor associated peptides may find antigen-naïve T cells, or antigen pre-exposed T-cells which may exhibit responses comparable to those of convalescents [43].

### 3.7. Defining a Responding Peptide and Comparing the Sensitivity of FCM to A-FCM

We defined a peptide as “responding” if it had fulfilled two criteria. (1) The peptide demonstrated an increase in net response (i.e., peptide minus background) at one or more time points (e.g., t1, t2 and booster) compared to net baseline response (t0). (2) The peptide that passed criterion #1 at a specific time point should also be *higher*, at that time point, from the background level plus 1.65 standard deviations (STD) (1 sided *t*-test with α = 5%). For FCM, the mean background + 1.65STD for CD4 responses was 1.73%, and for CD8 it was 0.033%. Differently, for A-FCM, the mean CD4 background + 1.65STD was 0.046% and for CD8 responses it was 0.021%.

We compared the number of responding peptides between A-FCM and FCM when the used functional marker/s was only IFNγ (Table 1) or were IFNγ and/or CD107a (Table 2). Both Table 1 and Table 2 show that FCM entirely missed the responses of CD4+ T cells to peptide vaccines, compared to A-FCM, owing to the above criterion #2 (background + 1.65STD). The differences between the approaches were less pronounced for CD8+ T cells.

We expected a strong overlap between peptides denoted as “responding” between FCM and A-FCM, at least in the CD8 T-cell subset. We found that the addition of CD107a to IFNγ (Table 2) enhanced the contingency between FCM and A-FCM (79% overlap) compared to the use of IFNγ alone (64% overlap) (Table 2). Statistically, the contingency between A-FCM and FCM for IFNγ+CD107a was significant (*p* = 0.0027, Fisher’s exact) while it was not significant for IFNγ alone (*p* = 0.14, Fisher’s exact). This underscores the importance of the addition of CD107a to the set of functional markers, in addition to its ability to enable monitoring of polyfunctionality [4].

### 3.8. Comparing A-FCM to ELISpot

Since the full A-FCM approach is more sensitive than the ELISpot approach, we defined the peptides it had identified as “ground true” for the sake of this assay. We then compared “true” peptides identified by A-FCM in set-2 with the peptides identified by ELISpot for the same set. ELISpot identified only one of eight true peptides in set-2, with a calculated sensitivity of 12.5% (true positive/all positive) and specificity of 22.2% (true negatives/all negatives). Interestingly, examination of the sole peptide identified by ELISpot in this set revealed that it was also the peptide which had exhibited the strongest response in the A-FCM approach (S2P19 d9). Similar low-sensitivity and low-specificity of ELISpot were observed with the CRC and the HD samples (overall sensitivity 33%, specificity 83%).

## 4. Discussion

Accurate detection of T-cell responses in personalized peptide/RNA vaccines is critical for monitoring an individual patient’s treatment efficacy and for optimization of vaccination strategy (e.g., how many peptides to use per individual set, timing of vaccinations, dosages, etc.). Both ELISpot and standard FCM approaches are limited in their ability to detect weak, albeit physiologically responding, T cells due to limited assay sensitivity as well as to antigen-non-specific background IFNγ-secretion. ELISpot suffers from low sensitivity and high variability, while standard FCM suffers from high background activation noise and high variability.

Several additional methodologies have been developed to sensitively identify antigen-specific T-cell responses. Tetramers (or MHC-multimers) are an FCM based approach to detect antigen-specific cells based on the binding of fluorescently labeled peptide-loaded-MHC multimers to antigen-specific T cell receptors. The main limitation in using tetramers is the requirement for knowledge of the peptide and the MHC allele involved in the predicted binding. This makes monitoring of peptide pool-stimulated T cells largely impractical. Moreover, there is a limited spectrum of available tetramers: from thousands of known HLA genotypes, approximately a hundred HLAs class I and II tetramers are readily available [44]. In the case of the glioma patient, tetramers were available in the large NIH tetramer core facility [45] for only 2/6 of the HLA-class I alleles and for 3/7 of the HLA-II allele [46]. Additionally, the affinity required for staining with tetramers is higher than that required for efficient T cell activation, thereby missing fully functional low-affinity T cells [44]. This may be important when monitoring responses to non-mutated tumor antigens, as CTA, which utilize lower affinity T cell receptors [46]. Lastly, a single long peptide (20aa) may bind to several MHC class-II alleles and may also encode several additional MHC-I epitopes. This increases the complexity and cost of each assay. Practically, with limited blood samples and the low frequencies of tumor-antigen specific T cells (requiring monitoring of larger cell numbers) the analysis of multiple samples per a single long peptide may likely reduce the number of aptly monitored peptides.

An additional methodology developed by Dimitrov et al. for quick and sensitive detection of T cells uses changes in the conformation and clustering of β2 integrins occurring minutes after antigen encounter [47]. The assay monitors only CD8+ T cells and has some aspects limiting its wide use: The first is that different antigens show considerably different activation dynamics throughout relevant assay timelines (8–120 min), with changes ranging from 1.5 to over 10 folds. The second limitation is that during this limited assay duration, the assay more prominently identifies cells with early and high functional capabilities (e.g., cells strongly secreting IFNγ within 1–2 h of antigen encounter). Notably, sixteen-hour ICS assays were found to identify more functionally responsive antigen specific T cells than 0, 1 or 6 h assays [48].

The A-FCM approach allows increased sensitivity for the detection of both weak and strong T-cell responses. The method uses a tight eight-color panel which reduces non-specific responses/background by means of several parallel approaches. These include the gating out of dead and unwarranted cells, the accurate identification of live T cells and the important addition of two early activation markers. The recently activated T cells are monitored for their polyfunctional responses to various antigens.

The main novelty of our panel is the addition of two early activation markers using an OR logical gate upstream to the polyfunctional analysis. Others have also used an activation marker together with a functional marker [19,26,36]. For instance, OMIP-022 [34] uses CD107a and CD40L in a single panel, yet CD40L that was used as a single activation marker, was part of the polyfunctional array. CD40L, in that case, was not used upstream of polyfunctionality, a strategy which enabled us to identify cells responding within the assay well.

Thieme et al. [32] have monitored IFNγ secretion in response to viral peptides using activation-marker double-positive (CD40L+4-1BB+) CD4 T cells (using an AND logical gate) and single positive (4-1BB+) CD8 cells. As our data shows (and that from Thieme’s), many of the recently activated CD4 T cells were either CD40L+4-1BB− or CD40L−4-1BB+ but not double positive. In our data, focusing only on the CD40L+ 4-1BB+ Function+ CD4 T cells, or the 4-1BB+ Function+ CD8 T cells in the CRC/HD sample set would bring about the loss of approximately 72% and 27% of the identified Activation+ Function+ CD4 and CD8 T cells, respectively. Taken together, the inclusion of the two activation markers, upregulated by either CD4 or CD8 T cells [19,32,33,49] powers the ability to sensitively detect rare antigen-specific T cells.

There are several other potential T-cell activation markers that could be used, such as CD69 and CD25; however, their constitutive or non-exclusive expression on some T cells restrict their use in our case [19]. 4-1BB and CD40L were selected due to their negligible expression prior to antigenic activation and their dependency upon TcR activation [36,37]. 4-1BB ligation provides co-stimulation and anti-apoptotic functions, promoting T-cell proliferation and survival [36,50]. CD40L interacts with its receptor, CD40, which is expressed on APC, and is then internalized and degraded [26,37]. These markers in combination satisfactorily identify recently activated CD8 and CD4 T cells [19,26].

FCM as a monitoring methodology has advantages and disadvantages. On the one hand, it provides rich multiparametric information on the single-cell level; on the other hand, FCM harmonization is challenging, limiting its wide clinical utility [22]. That said, multicolor FCM can provide critical diagnostic data conforming to the highest clinical standards, as in the case of the diagnosis of hematological malignancies by the Euroflow panels [51]. In addition, the significant advantage of FCM (whether using A-FCM or not) is the ability to choose what to focus on within all the parameters contained in the panel (e.g., focusing only on IFNγ while monitoring also CD107a).

The advantage of ELISpot is that it is an easily-performed technique that does not require high technical skills or expensive lab instrumentation [15]. The assay can also be easily harmonized across laboratories [15]. Its disadvantages are that it is limited in sensitivity in detecting individual positive cells compared to FCM. In our assays that utilized strong antigens (CEFT, SEB), IFNγ ELISpot had identified several-fold fewer cells than the numbers identified by IFNγ ELF or FCM (Figure 3c,d). The assay’s lower sensitivity, which limited the number of acquired positive cells, frequently drove ELISpot to base its results upon only a handful of cells [13]. The rare cells/SFUs are Poisson-distributed with a small λ (representing both the Poisson mean and its variance), thereby generating a large CV. This means that any haphazard low or high numbers of events in the negative control samples may randomly assign peptides to be positive or negative. While the paucity of assayed cells is a limit to accuracy in FCM as well, the increased per-cell sensitivity of FCM ameliorates this problem.

Others have also compared ELISpot to FCM. Hagen et al. compared IFNγ FCM of T cells (gated solely on CD3) to IFNγ ELISpot and to IFNγ ELISA on PBMC activated by phorbol myristate acetate (PMA)+ionomycin. Similarly to some of our data, those authors identified low and sometimes opposite correlations between the three different methodologies [29]. While ELISpot and FCM generally correlated, significant discrepancies were found in within-donor comparisons [29]. Hagen et al. also found that FCM detected 2–3 times more positive cells than ELISpot.

Karlsson et al. compared FCM and ELISpot using viral peptide pools on SEB-stimulated PBMC [52]. The calculated Pearson correlations between the two methodologies (ranging from 0.55 to 0.72) for these strong antigens indicated “*a considerable lack of numeric agreement*”. Their findings that different cell concentrations used in ELISpot produced substantial and sometimes contrasting effects on the frequencies of detected SFUs were also worrisome. Again here, ELISpot tended to underestimate the frequency of IFNγ-secreting T cells [52].

Our study confirms and extends the above-cited studies’ findings that had been obtained using Hagen’s two-color panel [29] or Karlsson’s four-color panel [52], with several differences. We evaluated responses to both weak and strong antigens (peptide pools, CEFT, CTA, neoantigens, and other TAAs). We included both CD4 and CD8 T-cell markers, which enabled dissection of the response of these two distinct T-cell subpopulations. We used a viability dye, which is critical for correct enumeration of responding T cells, especially in ICS assays [14] and when working on rare cells [19]. Lastly, our use of two early activation markers enabled the detection of T-cell responses in frequencies that are several orders of magnitude lower than those found to viral antigens or to superantigens [1,19,53]. In summary, our data, together with those of others, suggest that while ELISpot may be a valid preference for the detection of responses to strong antigens. Many, possibly even most, of the responses to weak antigens (e.g., tumor associated antigens) may not be accurately detected by ELISpot or may be incorrectly classified as positive or negative.

The A-FCM approach can be used for preclinical research as well as for clinical applications. Kallas et al. have demonstrated, in an HIV vaccine trial that T cell–based vaccine strategies should aim at compiling the smallest set of relevant vaccine antigens. This reduce interference between individual vaccine components, driving the induction of the maximally achievable immune response [54]. 

The A-FCM approach has already been put to use in a compassionate use clinical trial with a child suffering from glioma. Identification of the peptides that had succeeded in eliciting a response enabled the assembly of a compacted vaccine composed solely of those peptides. We further use this methodology to compare differences in responses to self-antigens [55] in CRC patients versus HDs. Note that while IFNγ cellular responses were previously correlated with clinical benefit [12], future work will be required to establish whether the frequencies of Activation+ Function+ T cells also correlate with clinical responses.

The eight-color panel can be easily adopted by labs proficient in multicolor FCM. Possible extensions to this panel can be achieved by adding antibodies to cytokines of additional polarizations (e.g., TGFβ for T_reg_, IL17A for Th_17_, etc. [56,57,58]). Additional activation markers can be added to the panel, possibly identifying additional activated cells. Such activation markers should demonstrate a near zero background, TcR-dependency, and should be compatible with the assay’s timelines. Tetramers can also be used to further focus the identification of TcR-specific cells in patients restricted to specific HLA alleles (e.g., HLA-A0201). The addition of markers, such as CD45RA, CCR7 and others, can be used to evaluate T-cell maturation states [59].

New approaches and instrumentation are changing the field of immune monitoring. Harmonized multicolor FCM is standardly used for immunophenotyping and diagnosis of hematological malignancies [51]. New devices can now automate and standardize staining procedures, sample acquisition and sample analysis reducing the likelihood of technical man-made errors [60]. These technological developments, together with better immune-monitoring protocols, may help push forward the rapidly evolving field of personalized cancer immunotherapy.

## 5. Conclusions

Current methodologies used for the detection of T cells activated in response to specific antigens, suffer from low sensitivity, thereby missing the detection of many responding peptides. Our methodology contributes to a set of known sensitivity-enhancing measures an additional critical measure for enabling the detection of T cells that have responded to antigens within the assay well. This reduces the non-specific T-cell response background by more than ten-fold and makes it possible to detect many, otherwise undetectable, T-cell responses. Our methodology provides an important monitoring tool for application in the field of personalized antigen-specific cancer immunotherapy.

## Figures and Tables

**Table 1:** Identification of peptides by using single function (IFNγ) to three vaccine sets. CD4+ and CD8+ T cells responses to peptides in 3 sets of glioma vaccine (S1–3) and their booster were analyzed by activation marker-inclusive FCM (A-FCM) and FCM. Overlapping peptides are those identified both by A-FCM and by standard FCM (FCM).

IFNγ Only	Peptides Identified by A-FCM (P1–P30)		Peptides Identified by FCM (P1–P30)		% Overlap between the Two Analyzed Methods	
	#	%	#	%	# (overlap)	% (overlap)
CD4	10	33	0	0	0	0
CD8	14	47	14	47	9/14	64
Total # peptides	15	53	14	47	9/15–9/14	60-64

**Table 2:** Identification of peptides by using dual-function (IFNγ and CD107a) to three vaccine sets. CD4+ and CD8+ T-cell responses to peptides in 3 sets of glioma vaccine (S1–3) and their booster were analyzed by activation marker-inclusive FCM (A-FCM) and standard FCM. Overlapping peptides are those identified by both A-FCM and the FCM approaches.

IFNγ and/or CD107a	Peptides Identified by A-FCM (P1–P30)		Peptides Identified by FCM (P1–P30)		% Overlap between the Two Analyzed Methods	
	#	%	#	%	# (overlap)	% (overlap)
CD4	13	43	0	0	0	0
CD8	14	47	14	47	11/14	79
Total # peptides	18	60	14	47	11/18–11/14	61–79

## Data Availability

The data presented in this study are available upon reasonable request from the corresponding author.

## Supplementary Materials

The following supporting information can be downloaded at https://www.mdpi.com/article/10.3390/cancers15030574/s1, Table S1: antigens used for the glioma vaccine, Table S2: assay reproducibility, Figures S1 and S2: timeline of vaccination or blood sampling; Figure S3: Secretion of IFNγ by vaccination-targeted and untargeted cells for CRC and HD; Figure S4: flow cytometry with early activation markers (A-FCM) for CRC and HD.
