# Supplementary material for: A Highly Sensitive Flow Cytometric Approach to Detect Rare Antigen-Specific T Cells: Development and Comparison to Standard Monitoring Tools
Source: Cancers (Basel). 2023 Jan 17;15(3):574. doi: 10.3390/cancers15030574 (PMC9913544; doi:10.3390/cancers15030574)
Supplement: Supplementary file 1 [file cancers-15-00574-s001.zip › cancers-2098111-supplementary.doc]

A Highly Sensitive Flow Cytometric Approach to Detect Rare Antigen-Specific T Cells: Development and Comparison to Standard Monitoring Tools

Meytal Dror Levinsky, Baruch Brenner, Michal Yalon, Zohar Levi, Zvi Livneh, Zoya Cohen, Tamar Paz-Elizur, Rachel Grossman, Zvi Ram and Ilan Volovitz

Supplementary materials:

**Table S1. Antigens used for glioma vaccine and their capacity to activate T cells at three time points.** + refers to activation of either CD4+ and/or CD8+T cells, - denotes no activation found in either subset.

| **#** | **Length** | **Target** | **Day 9-14** | | **Day21-22** | **Booster** |
| --- | --- | --- | --- | --- | --- | --- |
| S1P1 | 20 | KDM5B | + | - | | - |
| S1P2 | 20 | HSPA1A | - | - | | - |
| S1P3 | 20 | MGAT5 | - | - | | - |
| S1P4 | 20 | PRAME | + | + | | - |
| S1P5 | 20 | GPATCH2 | - | - | | - |
| S1P6 | 14 | NF1 | - | - | | - |
| S1P7 | 16 | CUL7 | - | - | | - |
| S1P8 | 9 | H3-3A G35R | - | - | | - |
| S1P9 | 9 | TP53 R248Q | - | - | | - |
| S1P10 | 12 | CUL7 | + | - | | - |
| S2P11 | 20 | SPAG9 | + | + | | + |
| S2P12 | 20 | AKAP13 | + | - | | + |
| S2P13 | 20 | SCRN1 | + | - | | - |
| S2P14 | 20 | SART3 | - | + | | + |
| S2P15 | 20 | DCAF12 | - | - | | + |
| S2P16 | 20 | CSF1 | + | + | | - |
| S2P17 | 20 | HAGE | - | - | | + |
| S2P18 | 20 | DDX53 | + | - | | + |
| S2P19 | 18 | TP53 R248Q | - | - | | + |
| S2P20 | 20 | H3-3A G35R | + | - | | - |
| S3P21 | 27 | H3.3 G34R | + | - | | - |
| S3P22 | 20 | CNOT3 | - | - | | - |
| S3P23 | 27 | SBSN | - | - | | - |
| S3P24 | 27 | PABPC3 | - | - | | - |
| S3P25 | 27 | GPR32 | - | - | | + |
| S3P26 | 27 | CCT8L2 | - | - | | + |
| S3P27 | 27 | KIAA2013 | + | - | | - |
| S3P28 | 27 | CLIC6 | - | - | | + |
| S3P29 | 27 | USP17L10 | - | - | | - |
| S3P30 | 27 | TSSK2 | - | - | | - |

**Table S*1*. A reproducibility assay for the activation-marker inclusive FCM (A-FCM) approach.** A-FCM was tested for it inter- and intra-assay reproducibility using a healthy donor's PBMC sample. The PBMC sample was cultured with various self-antigen peptide pools (MUC1 or CEA), with a viral peptide pool (CEFT) or without peptides, and stained using the A-FCM panel. Two assays were run in triplicates in two different time points. Net response was calculated for each well by subtracting mean of no-peptide from the antigen specific response. The standard deviations (STD) for triplicates of cells cultured with a specific antigen, representing intra-assay noise, were small (average of mean STDs for CD4+ and CD8+ T cells = 0.015%). The means of the absolute differences between triplicate repeats, representing inter-assay noise, were also small (average of triplicate differences for CD4+ and CD8+ T cells = 0.027%).

| **T cells subset** | ∆Calculation = (Antigen) – (Mean of no peptide) | _∆Actin_ | | _∆CEFT_ | | _∆MUC1­_ | | _∆CEA_ | |  |
| --- | --- | --- | --- | --- | --- | --- | --- | --- | --- | --- |
|  |  | Repeat 1 | Repeat 2 | Repeat 1 | Repeat 2 | Repeat 1 | Repeat 2 | Repeat 1 | Repeat 2 | Mean |
| **CD4** | STD of repeats | 0.0025 | 0.0080 | 0.0110 | 0.0018 | 0.0012 | 0.0042 | 0.0056 | 0.0044 | 0.0048 |
|  | │Repeat 1- Repeat2│ | 0.0004 | | 0.0129 | | 0.0009 | | 0.0080 | | 0.0055 |
| CD8 | STD of repeats | 0.0107 | 0.0057 | 0.0270 | 0.0738 | 0.0253 | 0.0215 | 0.0079 | 0.0238 | 0.0245 |
|  | │Repeat 1- Repeat2│ | 0.0167 | | 0.1079 | | 0.0562 | | 0.0163 | | 0.0493 |

**Figure S1. Timeline of Glioma patient’s injections and blood lettings**. The patient was administered three sets of vaccines S1-3, each with 10 different peptides (P1 to P30) and a booster vaccine with the same peptides per each set. Blood was let at or before the 1^st^ vaccination, at early time point 9-14d post injection (pi), and at later time point 21-22d pi. Peripheral blood mononuclear cells (PBMC) were purified from blood samples using the buffy layer method and frozen. T cell activation assays were performed by culturing thawed PBMC (in average 4.9x10^5^) cultured with single peptides (from the glioma vaccine) or with negative or positive controls (see text).

.

**Figure S2. Timeline of bloodletting from colorectal cancer (CRC) patients and healthy donors.** Healthy donors were considered those with normal colonoscopy, and no other diagnosed cancer. Peripheral blood mononuclear cells (PBMC) were purified from blood samples using the buffy layer method and frozen before use. T cell activation assays were performed by culturing thawed PBMC (in average 4.9x10^5^) cultured with various peptide pools (see text).

**Figure S3. Secretion of IFNγ by vaccination-targeted and untargeted cells:** CRC patient and HD PBMC were cultured with or without peptide pools overnight and stained using the T-cells polyfunctionality panel. **(a)** Cumulative IFNγ secretion by the cell subsets defined in the legend. ELISpot- like flow cytometry (ELF) analysis emulates what could be the results of an ELISpot assay using flow cytometric data (data of higher resolution). Responses were evaluated to self-antigen peptide pools, to positive control viral antigen peptide pool (CEFT) and to no peptides (negative control). **(b)** Similar analysis of IFNγ secretion to SEB - a superantigen stimulating polyclonal T cell responses.  *In most samples, the majority of IFN*γ *secretion originated from non CD4+ or CD8+ T cells.*

**Figure S4. The addition of early activation markers reduces non-specific activation background in T cells activation assays.** A colorectal cancer (CRC) patient and healthy donor (HD) PBMC samples were cultured with self-antigen peptide pools (MUC1 or CEA) or without peptides overnight and stained using a T-cell polyfunctionality panel, inclusive of the CD40L and 4-1BB activation markers (A-FCM). Net activation was calculated by subtracting the percent functionally active cells [IFNγ secretion and/or degranulation (CD107a)], which are also activation-marker positive (CD40L and/or 4-1BB positive), in the peptide non-stimulated samples, from the response in matched peptide-stimulated samples. Graphs show the mono- and polyfunctional responses of CD4+ and CD8+ T cells. The assay demonstrates that adding early activation markers reduces non-specific background responses. This reduction in background enables the identification of minute yet physiological T-cell responses.
